# Supplementary figures and images for: Assessing anti oxidant, antidiabetic potential and GCMS profiling of ethanolic root bark extract of Zanthoxylum rhetsa (Roxb.) DC: Supported by in vitro, in vivo and in silico molecular modeling
Source: PLoS One. 2024 Aug 19;19(8):e0304521. doi: 10.1371/journal.pone.0304521 (PMC11332921; doi:10.1371/journal.pone.0304521)

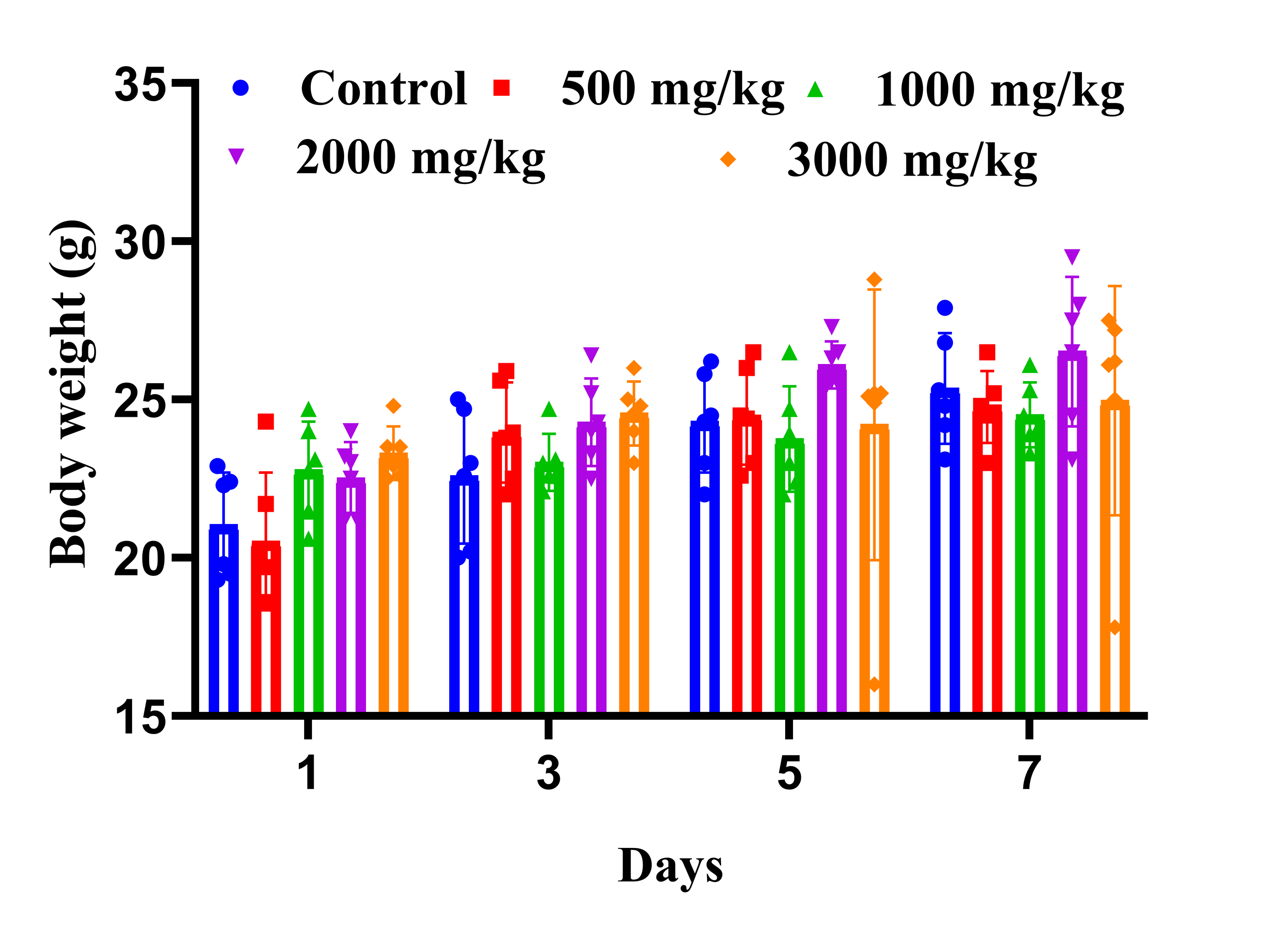

Supplement: S1 Fig — (TIF) [file pone.0304521.s001.tif]

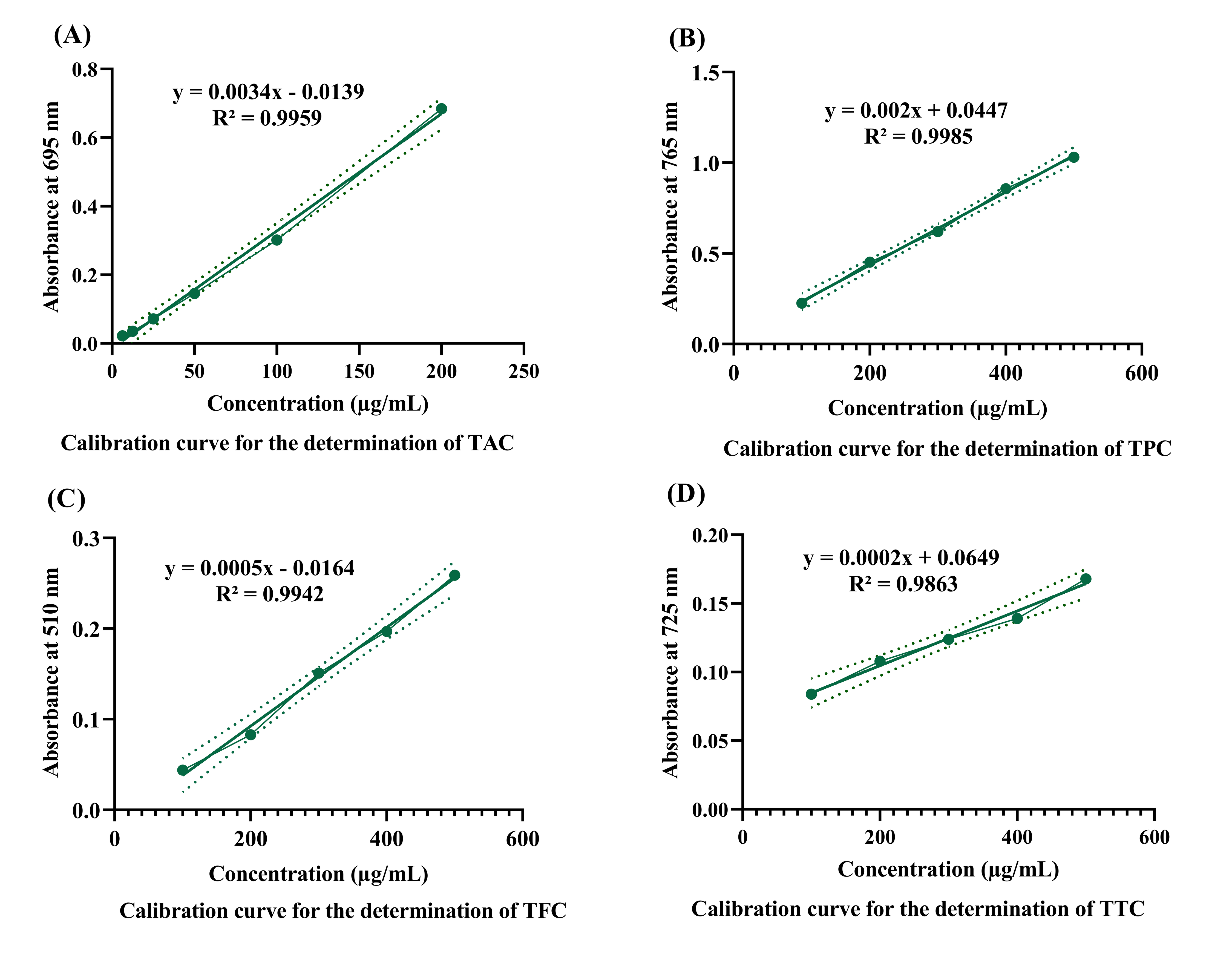

Supplement: S2 Fig — Standard calibration curves for the calculation of (A) total antioxidant activity (TAA); (B) total phenolic contents (TPC); (C) total flavonoid contents (TFC); and (D) total tannin contents (TTC) of ZRRB extract. (TIF) [file pone.0304521.s002.tif]

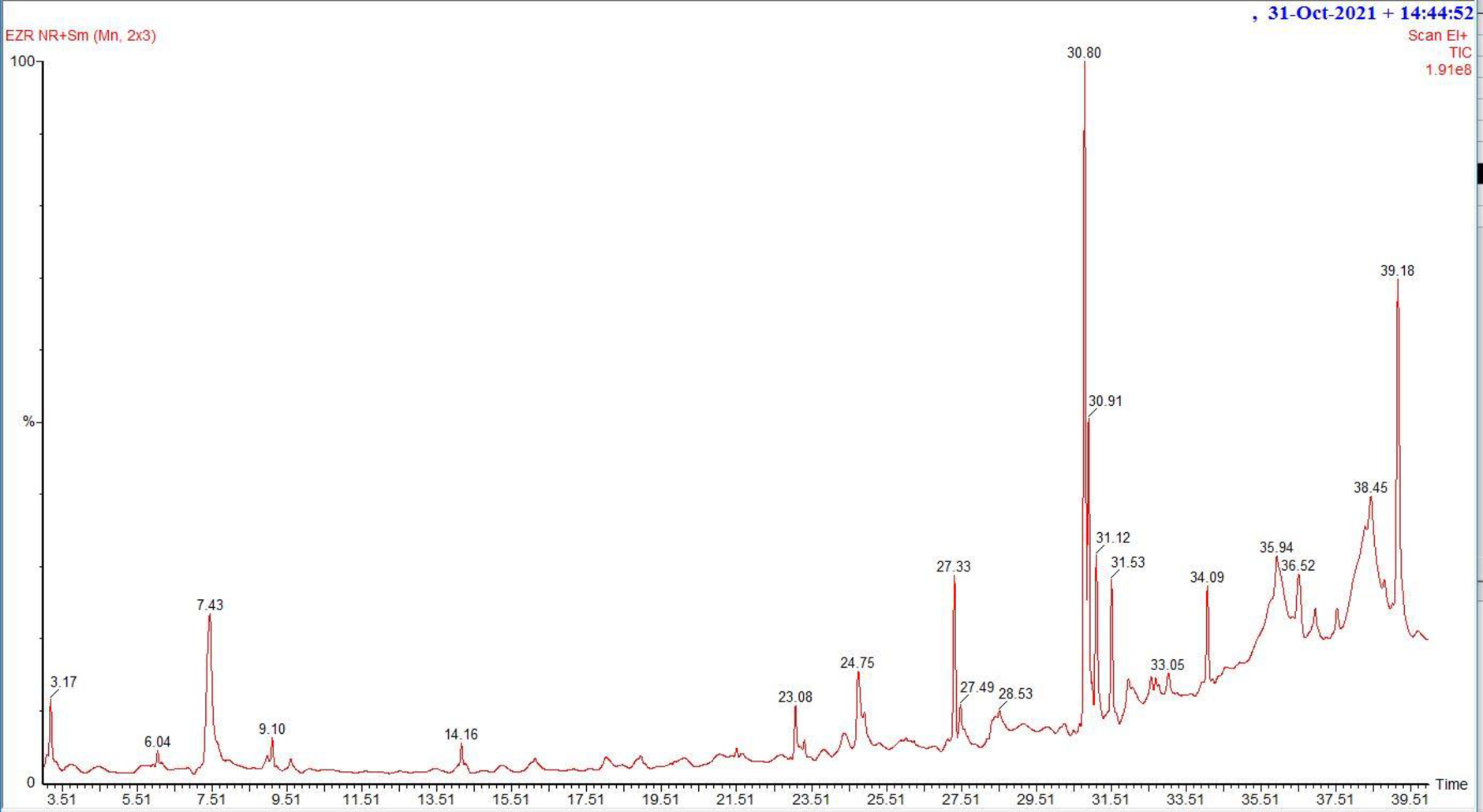

Supplement: S3 Fig — (TIF) [file pone.0304521.s003.tif]

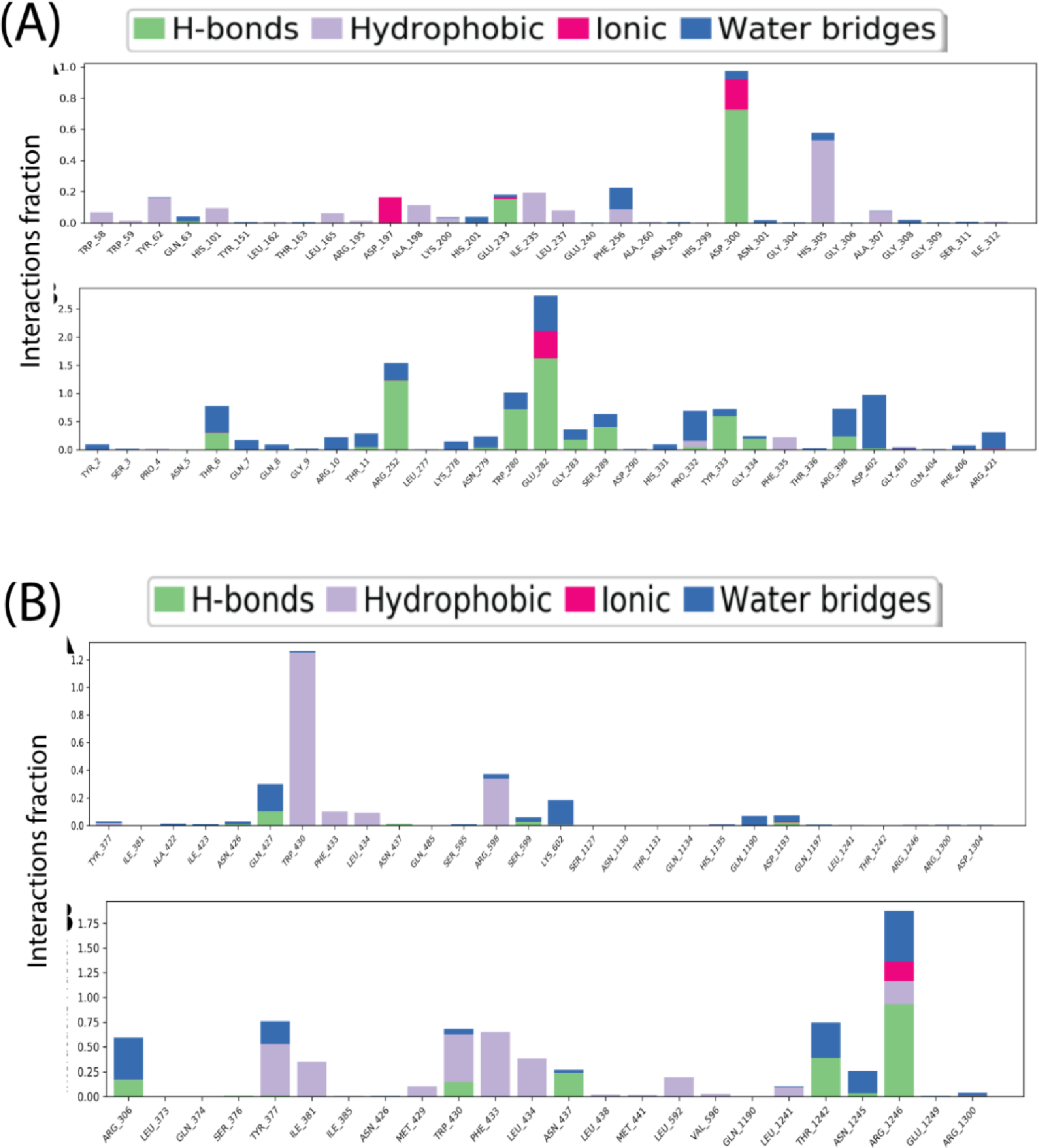

Supplement: S4 Fig — The figure expresses the interaction of ligands with the targeted two proteins. (A) α-amylase (PDB ID: 1HNY), where the selected compound (CID: 97176) and control drug (Acarbose); (B) Sulfonylurea receptor 1 (PDB ID: 5YW7), where the selected compound (CID: 97176) and control drug (Glibenclamide). The selected ligand and control drugs indicate blue and orange color, respectively. (TIF) [file pone.0304521.s004.tif]
