# Supplementary material for: Assessing anti oxidant, antidiabetic potential and GCMS profiling of ethanolic root bark extract of Zanthoxylum rhetsa (Roxb.) DC: Supported by in vitro, in vivo and in silico molecular modeling
Source: PLoS One. 2024 Aug 19;19(8):e0304521. doi: 10.1371/journal.pone.0304521 (PMC11332921; doi:10.1371/journal.pone.0304521)
Supplement: S1 Table — (PDF) [file pone.0304521.s005.pdf]

S.NO	Compounds name	Binding affinity (kcal/mol)		
		**Peroxiredoxin 5**	**α-Amylase**	**SUR 1**
1	3-(1'-pyrrolidinyl)-2-butanone	-4.2	-4.5	-5.1
2	Dl-threitol	-4.2	-4.4	-3.9
3	Trans-2,4-dimethylthiane, s,s-dioxide	-4	-4.3	-4.5
4	Chloroacetic acid, tetradecyl ester	-4.5	-4.9	-4.8
5	2,4-difluorobenzoic acid, 2-propylphenyl ester	-6.1	-7.4	-7.6
6	3',5'-dimethoxyacetophenone	-5.2	-5.2	-5.7
7	Beta.-l-arabinopyranoside, methyl	-5.1	-5.6	-4.9
8	Methyl 11-methyl-dodecanoate	-4.1	-4.7	-5.4
9	(E)-4-(3-hydroxyprop-1-en-1-yl)-2-methoxyphenol	-5.3	-6	-5.9
10	Dibutyl phthalate	-5.2	-5.6	-6.1
11	13-octadecenoic acid, methyl ester	-4.3	-4.8	-5.2
12	12,15-octadecadienoic acid, methyl ester	-4.6	-5	-5.6
13	Methyl 8,11,14-heptadecatrienoate	-4.9	-5.6	-5.4
14	2H-1-benzopyran-2-one, 3,4,7-trimethoxy	-5.7	-5.9	-5.9
15	Hentriacontane	-4.4	-5	-5
16	6H-indolo[3,2,1-de][1,5]naphthyridin-6-one	**-6.8**	**-7.7**	**-8.1**
17	Ascorbic acid (Standard)	-5.6		
18	Acarbose (Standard)		**-7.7**	
19	Glibenclamide (Standard)			**-8.5**
