# Supplementary material for: Assessing anti oxidant, antidiabetic potential and GCMS profiling of ethanolic root bark extract of Zanthoxylum rhetsa (Roxb.) DC: Supported by in vitro, in vivo and in silico molecular modeling
Source: PLoS One. 2024 Aug 19;19(8):e0304521. doi: 10.1371/journal.pone.0304521 (PMC11332921; doi:10.1371/journal.pone.0304521)
Supplement: S2 Table — (PDF) [file pone.0304521.s006.pdf]

Properties	Model Name	Compounds				
		1549095	91717595	606454	95997	97176
**Absorption**	Intestinal absorption (human)	91.734	94.591	97.815	96.413	100
	Skin Permeability	-2.848	-2.338	-2.611	-2.307	-2.323
	caco-2	1.227	1.716	1.322	1.445	1.623
**Distribution**	VDss (human)	-0.125	-0.126	-0.476	-0.137	0.137
	Fraction unbound (human)	0.292	0.002	0.287	0.385	0.319
	BBB permeability	-0.182	0.344	0.085	0.421	0.856
	CNS permeability	-2.569	-1.353	-2.437	-2.192	-1.656
**Metabolism**	CYP2D6 substrate	No	No	No	No	No
	CYP3A4 substrate	No	Yes	No	Yes	Yes
	CYP2D6 inhibitor	No	No	No	No	No
	CYP3A4 inhibitor	No	No	No	No	No
**Excretion**	Total Clearance	0.233	0.345	0.79	0.723	0.35
**Toxicity**	AMES toxicity	No	No	No	No	Yes
	Hepatotoxicity	No	Yes	No	No	No
	Skin Sensitization	No	No	No	Yes	No
**Physicochemical**	Molecular weight	180.2	276.28	236.22	180.2	220.23
	Num. H-bond acceptors	3	4	5	3	2
	Num. H-bond donors	2	0	0	0	0
	Molar Refractivity	51.02	72.34	61.96	49.62	67.66
**Lipophilicity**	Consensus Log Po/w	1.62	4.55	1.89	1.80	2.39
**Water Solubility**	Log S (ESOL)	-2.23	-4.69	-2.64	-2.08	-3.43
	Solubility class	soluble	Moderately soluble	Soluble	Soluble	Soluble
**Drug-likeness**	Lipinski violation	Yes; 0 violation	Yes; 1 violation: MLOGP>4.15	Yes; 0 violation	Yes; 0 violation	Yes; 0 violation
	Bioavailability Score	0.55	0.55	0.55	0.55	0.55
**Medicinal Chemistry**	PAINs	0 alert	0 alert	0 alert	0 alert	0 alert
