# Supplementary material for: Assessing anti oxidant, antidiabetic potential and GCMS profiling of ethanolic root bark extract of Zanthoxylum rhetsa (Roxb.) DC: Supported by in vitro, in vivo and in silico molecular modeling
Source: PLoS One. 2024 Aug 19;19(8):e0304521. doi: 10.1371/journal.pone.0304521 (PMC11332921; doi:10.1371/journal.pone.0304521)
Supplement: Attachment — Submitted filename: Comments letter.docx [file pone.0304521.s007.docx]

**Comments**

1) The work is very interesting and studied for pre-clinical aspects. It will be useful for further research in the development of clinical studies.
2) It would be better to mention as anti-diabetes in the keywords instead of other words.
3) In silico should be in italics. In some instances, it is missing. It has to be checked and correct them.
4) In vivo should be corrected into italics.
5) References should be rechecked again according to the journal guidelines.

**Report**

The manuscript is well written and finds significance through in vitro, in vivo and in silico studies. My decision is ‘minor comments or revisions’. Therefore, I would strongly recommend the manuscript to be published in the Plos One Journal, after the above comments are addressed.
